# Study of Molten Pool Evolution in VP-CMT Aluminium Alloy Arc Additive Manufacturing Under Different EP:EN Ratios

**DOI:** 10.3390/ma19061237

**Published:** 2026-03-20

**Authors:** Xulei Bao, Yongquan Han, Fubiao Han, Lele Liu

**Affiliations:** 1 School of Materials Science and Engineering, Inner Mongolia University of Technology, Hohhot 010051, China; nmggydxbxl@163.com (X.B.);; 2 Institute of Water Conservancy in Pastoral Area, China Research Institute of Water Resources and Hydropower, Hohhot 010020, China

**Keywords:** VP-CMT, molten pool evolution, numerical simulation, Marangoni forces, aluminium alloy additive manufacturing

## Abstract

This study investigates the influence of varying positive–negative polarity ratios (EP:EN) on melt pool evolution during alternating current CMT (VP-CMT) arc additive manufacturing through a combined experimental and numerical approach. A multi-layer single-track droplet-melt pool coupling model was established, revealing the regulatory mechanisms governing melt pool flow, temperature distribution, and dimensional changes. These are driven by differences in arc morphology, heat input, and mechanical forces during EP and EN phases. Results indicate that molten pool flow is primarily governed by wire feed, retraction, and Marangoni forces. During the EP phase, arc divergence and elevated heat input result in significantly higher flow velocities than in the EN phase. Molten pool length increases with rising EP proportion, exhibiting periodic dynamic variations. Lateral flow intensity intensifies as EP ratio increases, directly influencing cladding layer morphology. This study provides theoretical basis for optimising additive manufacturing quality by adjusting the EP:EN ratio.

## 1. Introduction

**Figure 1:**
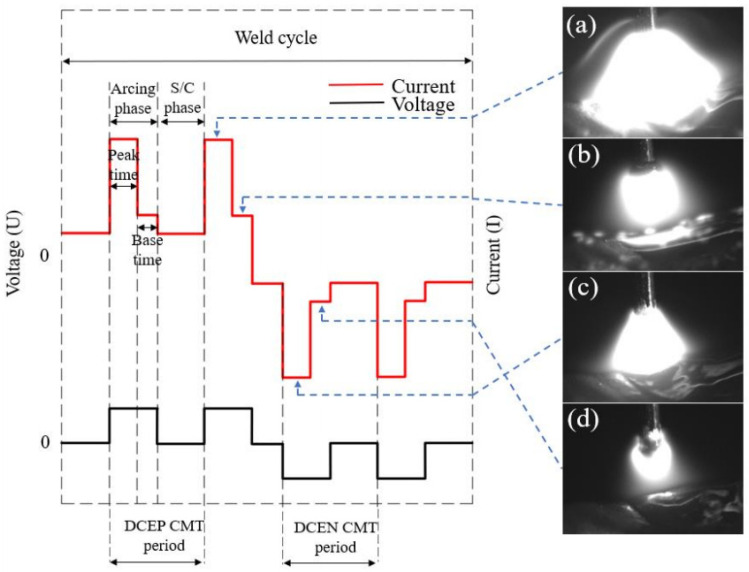
Typical AC CMT waveform and arc morphology [1]. (**a**) EPpeak period; (**b**) EPbase period; (**c**) ENpeak period; (**d**) ENbase period.

Cold Metal Transfer (CMT) technology combines droplet transition with wire retraction. During droplet transition, the arc extinguishes and the welding (additive) current drops to near zero, substantially reducing arc heat input. This process is highly suitable for additive manufacturing research on low-melting-point metals (aluminium alloys). The distinction between AC CMT and DC CMT lies in the introduction of a reverse-polarity arc. The entire AC CMT (VP-CMT) waveform alternates between positive-polarity phase EP and reverse-polarity phase EN, as illustrated in Figure 1. This depicts a typical AC CMT waveform and arc morphology. A single positive-polarity droplet transition cycle comprises an arc-ignition phase and a short-circuit phase, with the ignition phase further divided into peak and base stages. The EN phase cycle segmentation mirrors that of the EP phase. Compared to DC CMT, the inclusion of the EN phase further reduces heat input while enhancing thermal control, rendering it more suitable for arc additive manufacturing.

In arc welding and additive manufacturing, the arc plasma governs droplet transition and molten pool evolution through multi-field coupling (“electrical-magnetic-thermal-mechanical”), directly influencing formability and performance. However, conventional experimental methods struggle to quantitatively capture high-temperature arc, droplet, and molten pool internal physics. With advances in computational technology, research integrating numerical simulation with experimentation has become pivotal. This approach reveals the synergistic mechanisms of multi-physics fields, enabling a research breakthrough from empirical qualitative analysis to scientific quantification.

The evolution of the molten pool directly manifests arc-forming behaviour, and this region also exhibits the most complex multi-physics field dynamics during the forming process. Particular emphasis must be placed on elucidating the temperature field and flow field distribution patterns within the molten pool under the combined thermal effects of the arc plasma and molten droplets. This is especially crucial for enhancing forming quality and improving microstructural properties in arc welding and additive manufacturing processes. Consequently, the evolution of the molten pool constitutes a focal point in research concerning heat and mass transfer phenomena within arc welding and additive manufacturing processes. Concerning TIG molten pool evolution studies, Murphy [2] et al., Fan Ding et al. [3], and Wang Xinxin [4] primarily established weakly coupled arc-molten pool numerical models for computation. By calculating the temperature and flow field distributions within the molten pool, they sought to elucidate its evolution mechanisms under arc thermal effects. However, such weakly coupled models cannot provide an intuitive analysis of molten pool flow and free deformation phenomena.

To mitigate the impact of factors such as the high non-linearity and computational complexity inherent in welding and additive processes, researchers have modelled the thermal effects of arc plasma interaction with the molten pool, proposing heat source models and electrodynamic models. For instance, the Gaussian heat source model can accurately calculate the temperature field of the molten pool under conditions of low arc stiffness [5] and minimal impact force on the pool. Building upon the Gaussian heat source model, further developments include the three-dimensional conical Gaussian heat source model, the Gaussian cylindrical heat source model, the rotating Gaussian surface heat source model, and the rotating composite heat source model. However, due to differing heating ranges before and after the arc’s movement, Professor John Goldak [6] proposed the double ellipsoidal heat source model to address this issue. This model has been widely applied in numerical simulations of arc welding and additive manufacturing melt pool evolution behaviour. Simulation results obtained using the double ellipsoidal heat source model yielded significant penetration depth and a larger heat-affected zone, consistent with the morphology of the arc heat source melt pool. Furthermore, analytical expressions for the electromagnetic field within the molten pool were proposed by Kumar et al. [7] and Wu Chuansong et al. [8], enabling successful calculations of electromagnetic forces and arc pressure within the molten pool.

Zhou Xiangman et al. [9] at Three Gorges University established a three-dimensional weakly coupled model of droplet-melt pool interaction. Employing the CSF method to convert surface forces into volume forces, they analysed the temperature and flow fields within the melt pool under droplet disturbance. Their findings indicate that heat transfer by the droplet induces melt pool oscillation, with melt pool morphology significantly influencing the distribution of electromagnetic forces, heat flux, plasma shear forces, and arc pressure. Zhao et al. [10] employed a similar approach to simulate molten pool flow under droplet impact during MIG additive manufacturing. They observed droplet impact velocities reaching 0.9 m/s, driving downward flow of central molten pool metal to form a depression. Surface Marangoni forces propelled metal outward from the centre, while temperature gradients intensified flow at the pool’s leading edge, generating clockwise vortices at the pool bottom. Owen Min, Wei Yanhong et al. [11] developed an efficient three-dimensional numerical model for MIG arc additive manufacturing based on the minimum energy method for free-surface tracking. This model supports simulations of single-layer single-pass, multi-layer multi-pass, and non-linear path configurations, providing valuable insights for process design. Ogino et al. [12] investigated the effects of path strategy and interlayer temperature using a MIG molten pool model. Employing a Gaussian heat source distribution and arc pressure model, they simplified droplets as isothermal spheres, though simulated morphology dimensions deviated from experimental results. Bai et al. [13] developed a Particle-Aided Wave (PAW) model for additive heat and mass transfer, neglecting droplet impact by treating it as a source term with mass and temperature. A double ellipsoidal heat source was employed, with electromagnetic forces and arc pressure modelled using Gaussian distributions. Lei Yangyang and Xiong Jun [14] similarly simplified the molten droplet as an isothermal sphere, establishing a three-dimensional transient weakly coupled MIG model. They investigated the influence of process parameters on the heat and flow fields within the molten pool, achieving good agreement between simulated morphology and experimental results. Hashimoto et al. [15] developed a fixed-point model for short-circuit transition, identifying wire inclination angle, short-circuit current, and surface tension as key factors influencing spatter. However, this model disregarded wire motion. Cao et al. [16] developed a weakly coupled CMT model, observing symmetrical droplet flow when the substrate was horizontal and lateral deposition layer displacement when inclined. However, they simplified short-circuit transition to jet transition, neglecting liquid bridge effects. Zhao et al. [17] analysed the effects of different driving forces on molten pool flow in CMT, finding Marangoni forces exerted the most significant influence, with maximum molten pool velocities reaching 0.67 m/s. Increased substrate temperature intensified thermal accumulation, leading to greater weld width and depth but reduced height. He Junjie et al. [18] established a two-dimensional fixed-point CMT model based on a moving mesh, indicating that wire retraction and Marangoni forces dominate the behaviour of the magnesium alloy molten pool. Cadiou et al. [19] and Zhao Wenyong et al. [20] established strongly coupled arc–droplet-pool models, revealing pool evolution patterns under synergistic arc–droplet interactions. However, the former omitted Marangoni forces, while the latter simulated only half the domain to enhance efficiency. Experiments by Zhou et al. [21] revealed increased spatter with rising current in CMT+P processes, primarily due to droplet impact. However, quantitative analyses of temperature and flow fields were lacking, and few studies integrated heat and mass transfer behaviour to elucidate solidification mechanisms.

In summary, numerical simulations of heat and mass transfer in arc-based additive processes are transitioning from weakly coupled to strongly coupled models. Although research on melt pool evolution in arc welding and additive manufacturing has matured, studies on VP-CMT arc additive manufacturing melt pool behaviour remain unexplored.

## 2. Experimental Equipment, Materials and Research Methods

### 2.1. Experimental Materials

ER 5356 aluminium–magnesium welding wire possesses low density, high specific strength, and high fatigue limit, exhibiting excellent load-bearing capacity and the ability to withstand significant impact loads. The ER 5356 aluminium alloy welding wire was selected for testing with a diameter of 1.2 mm; its chemical composition is detailed in Table 1. The substrate for arc additive manufacturing must be selected with composition similar to the welding wire; otherwise, non-fusion between wire and substrate may occur, leading to cracking between the fabricated part and substrate. Additionally, substrate thickness must be appropriate: excessively thin substrates may deform, while excessively thick ones waste material. Therefore, an 8 mm thick 6061-T6 aluminium alloy substrate was selected. The chemical composition of 6061-T6 aluminium alloy is shown in Table 2.

### 2.2. Arc Additive Manufacturing System and High-Speed Camera System

**Figure 2:**
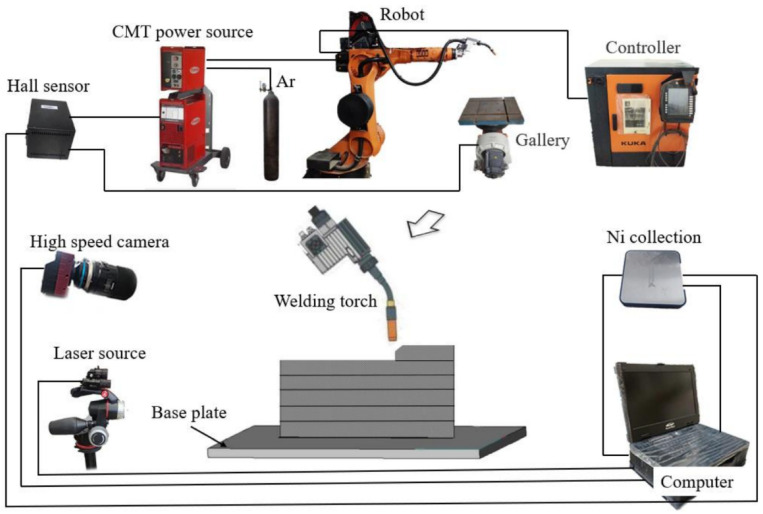
Schematic diagram of arc additive manufacturing system [1].

This experiment established an AC CMT additive manufacturing system and inspection system, as shown in Figure 2. The AC CMT additive manufacturing system primarily comprises a KUKA robot (Augsburg, Germany), Advanced 4000 welding power source (Pettenbach, Austria), and Spurt CAM offline programming software (SprutCAM 15). The high-speed camera system consists of a Rocke TECH AcutEye high-speed camera (Changsha, China), NI data acquisition system, and corresponding signal transmission circuitry. The additive manufacturing process employed in this experiment is detailed in Table 3.

## 3. Establishing a Multi-Layer Single-Pass Cladding Droplet Melt Pool Model

Considering that conventional computational parameters for multi-layer models would require excessive time for continuous additive layering up to 10 layers—approximately 24 h for a 2 s standard additive process—continuing beyond 10 layers would take several dozen days. This approach is impractical. Therefore, based on the deposition patterns observed in single-pass, single-layer melt pool models, the lower layers (below the 10th layer) are pre-generated using larger time steps. This enables rapid generation of the first nine deposition layers. For the 10th layer, precise transient multiphysics calculations with smaller time steps are performed near the midpoint.

Referencing high-speed camera imagery of droplet evolution and single-layer/single-pass droplet behaviour, we extracted parameters for droplet generation location, average droplet size and momentum, alongside momentum changes at the droplet-melt pool interface during retraction. These simulate wire feed and retraction processes:

Driving forces on the molten pool’s free surface comprise surface tension, Marangoni forces, and arc pressure. During CMT additive processes, these three forces critically influence the free surface morphology and flow patterns within the molten pool. Computational accuracy is intrinsically linked to interface tracking precision. Based on the VOF algorithm, the interface is determined by tracking cells with volume fractions between 0 and 1. This implicitly represented interface does not constitute the true boundary of the computational domain. Consequently, all forces acting on the free surface should be interpreted as continuous surface force (CSF) terms.

Surface tension constitutes a significant force influencing molten pool flow during welding, generally correlating with pool dimensions and surface temperature. Perpendicular to the molten pool surface, surface tension generates a pressure that affects surface morphology and the wetting of solid parent material by liquid metal. This surface tension pressure, *P_s_*, is determined by the following equation:

(1) $$P_{s}=\gamma \kappa$$

where γ is the surface tension coefficient, and κ is the surface curvature, which can be determined by the surface normal vector **n**.

(2) $$\kappa =\nabla \cdot \frac{n}{\left|n\right|}$$

Fluent employs the CSF (Continuum Surface Stress) model to calculate the pressure exerted by surface tension on the molten pool surface, converting it into volumetric force form. For two-phase flow, the volumetric force *F_vof_* corresponding to surface tension is as follows:

(3) $$F_{vol}=\gamma \frac{\rho \kappa \nabla \alpha_{i}}{\frac{1}{2}\left(\rho_{i}+\rho_{j}\right)}$$

where *α_i_* denotes the volume fraction of phase *i*, *ρ* is the fluid density at the interface, and $\rho_{i}$ and $\rho_{j}$ represent the densities of phases *i* and *j* respectively.

Moreover, surface tension is typically a function of temperature. A temperature gradient exists at the molten pool surface, where surface tension generates a shear force that induces surface flow—a phenomenon commonly termed the Marangoni effect. Marangoni shear force *τ_Ma_* is calculated by the following equation:

(4) $$\tau_{Ma}=\frac{d\gamma }{dT}\nabla_{s}T$$

where *T* denotes the molten pool surface temperature; $\nabla_{s}T$ represents the surface tangential vector.

The Marangoni force can be converted into a volumetric force using the continuous surface force (CSF) method:

(5) $$F_{ma}=\tau_{Ma}\nabla \alpha \frac{\alpha \rho_{\mathrm{metal}}+(1-\alpha )\rho_{\mathrm{gas}}}{\frac{1}{2}\left(\rho_{\mathrm{metal}}+\rho_{\mathrm{gas}}\right)}$$

The CMT arc pressure is the force exerted on the molten pool surface by the arc generated from the ionisation of argon gas. Previous arc force models were simplified to act on a plane surface. However, during arc additive manufacturing, the melting of the wire material causes accumulation on the base material surface, resulting in a molten pool shape that is not planar. Therefore, this paper employs the CSF method [13] to convert the arc force acting on a plane into a volumetric force that tracks the molten pool surface.

Arc force acting on a plane:

(6) $$F_{arcf}=\frac{\mu_{0}I^{2}}{\pi^{2}a_{f}b}\exp\left(-\frac{3{\left(x-x_{o}-vt\right)}^{2}}{a_{f}^{2}}-\frac{3y^{2}}{b^{2}}\right)\;\left(x\geq x_{o}+vt\right)$$

(7) $$F_{arcr}=\frac{\mu_{0}I^{2}}{\pi^{2}a_{r}b}\exp\left(-\frac{3{\left(x-x_{o}-vt\right)}^{2}}{a_{r}^{2}}-\frac{3y^{2}}{b^{2}}\right)\;\left(x\geq x_{o}+vt\right)$$

The continuous surface force (CSF) method enables conversion of the arc force into a volumetric force:

(8) $$F_{arcf}=F_{arcf}\nabla \alpha \frac{\alpha \rho_{\mathrm{metal}}+(1-\alpha )\rho_{\mathrm{gas}}}{\frac{1}{2}\left(\rho_{\mathrm{metal}}+\rho_{\mathrm{gas}}\right)}$$

(9) $$F_{arcr}=F_{arcr}\nabla \alpha \frac{\alpha \rho_{\mathrm{metal}}+(1-\alpha )\rho_{\mathrm{gas}}}{\frac{1}{2}\left(\rho_{\mathrm{metal}}+\rho_{\mathrm{gas}}\right)}$$

Improvements to the heat source model:

In additive welding simulations, the arc heat source is typically described using a double ellipse or double ellipsoid distribution. The surface heat source is more suitable than the volume heat source due to the use of the VOF method to track the molten pool’s free surface. This is because the volume heat source generally has a fixed spatial distribution, whereas the molten pool’s free surface is constantly changing. If the free surface traverses the spatial distribution of the volume heat source, part of the volume heat source will be applied to non-welded base material, as during the additive process.

According to the literature, the thermal model for the arc heat source can be expressed as follows:

(10) $$q_{arc}(x,y)=\left\{\begin{array}{c} (\frac{2a_{f}}{a_{f}+a_{r}})\frac{6\sqrt{3}UI}{\pi^{\frac{3}{2}}(af+ar)b}\exp(-\frac{3{\left(x-vt\right)}^{2}}{{a_{f}}^{2}}-\frac{3y^{2}}{b^{2}})\;\left(x-vt\geq 0\right)\\ (\frac{2a_{r}}{a_{f}+a_{r}})\frac{6\sqrt{3}UI}{\pi^{\frac{3}{2}}(af+ar)b}\exp(-\frac{3{\left(x-vt\right)}^{2}}{{a_{r}}^{2}}-\frac{3y^{2}}{b^{2}})\;\left(x-vt<0\right) \end{array}\right.$$

In FLUENT, incorporating the heat flux from the molten pool surface as a source term into the energy conservation equation necessitates its conversion into a volumetric term. When the molten pool surface undergoes significant deformation under the combined effects of arc pressure, droplet impact force, and evaporation back pressure—such as transitioning from a nearly planar surface to one with finite curvature—the planar heat source distribution model becomes inapplicable. Therefore, this heat source incorporates the VOF free-surface tracking method [22], proposing a curved surface heat source distribution that combines the welding heat source with the VOF-tracked surface:

(11) $$q_{arcx}=q_{arc}\nabla_{x}\alpha \frac{\alpha \rho_{\mathrm{metal}}+(1-\alpha )\rho_{\mathrm{gas}}}{\frac{1}{2}(\rho_{\mathrm{metal}}+\rho_{\mathrm{gas}})}$$

$$q_{arcy}=q_{arc}\nabla_{y}\alpha \frac{\alpha \rho_{\mathrm{metal}}+(1-\alpha )\rho_{\mathrm{gas}}}{\frac{1}{2}(\rho_{\mathrm{metal}}+\rho_{\mathrm{gas}})}$$

$$q_{arcz}=q_{arc}\nabla_{z}\alpha \frac{\alpha \rho_{\mathrm{metal}}+(1-\alpha )\rho_{\mathrm{gas}}}{\frac{1}{2}(\rho_{\mathrm{metal}}+\rho_{\mathrm{gas}})}$$

(12) $$Q_{arc}=q_{arc}\left|\nabla \alpha \right|\frac{\alpha \rho_{\mathrm{metal}}+(1-\alpha )\rho_{\mathrm{gas}}}{\frac{1}{2}(\rho_{\mathrm{metal}}+\rho_{\mathrm{gas}})}$$

Compared to the traditional double ellipsoidal heat source model, this heat source model demonstrates excellent adaptability to deformed free surfaces.

Model thermal boundary conditions:

The boundary conditions employed in this study are presented in Table 2. Convective and radiative boundary conditions are utilised to describe heat loss at the external boundary.

(13) $$-k\frac{\partial T}{\partial n}=h(T-T_{at})$$

(14) $$h=h_{con}+\varepsilon_{kb}\frac{T^{4}-{T_{at}}^{4}}{T-T_{at}}$$

**Figure 3:**
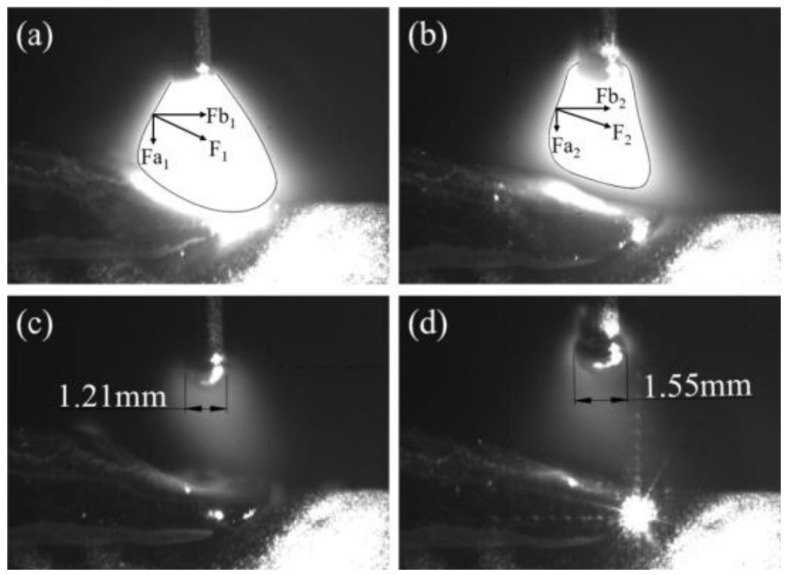
Arc morphology and droplet size during EP and EN periods. (**a**) EP period; (**b**) EN period; (**c**) Molten droplet size during the EP period; (**d**) Molten droplet size during the EN period.

**Figure 4:**
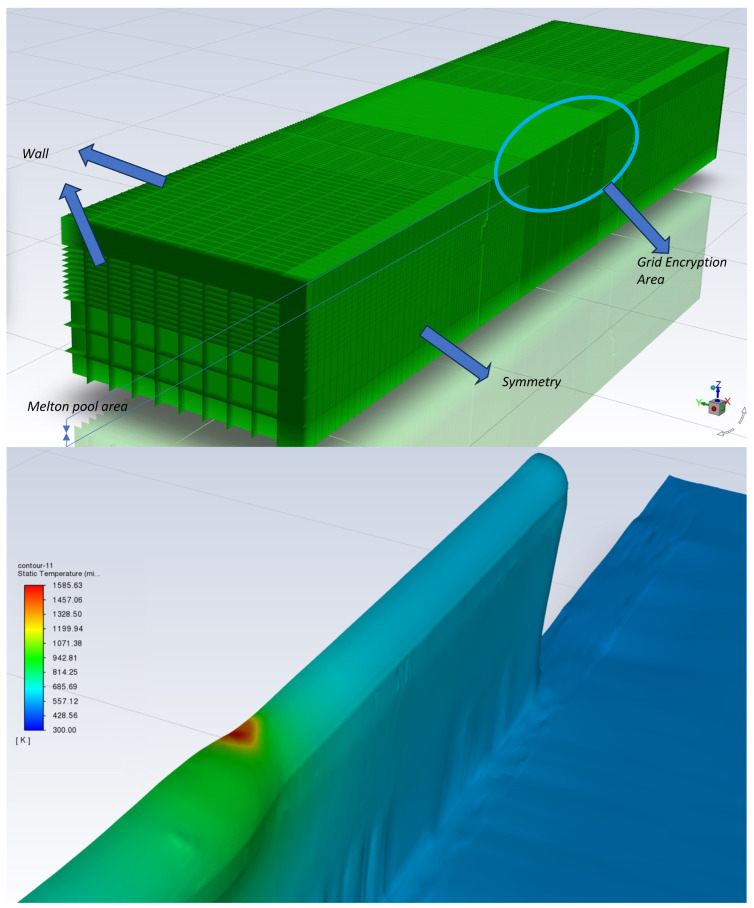
Mesh boundaries, mesh partitioning, and computational forming region in the multi-layer single-pass model.

The droplet volume size references the average droplet size in the high-speed camera model, refering to Figure 3 The momentum of the droplet prior to entering the molten pool references the droplet velocity upon initial contact with the molten pool in the droplet–molten pool model, as well as the velocity at the point of adhesion between the wire and molten pool during wire retraction. The grid boundaries, grid division and the computational forming area in the multi-layer single-channel model are shown in the Figure 4.

## 4. Study of Molten Pool Evolution Behaviour in EP/EN Stages of Multi-Layer Single-Pass VP-CMT Process

**Figure 5:**
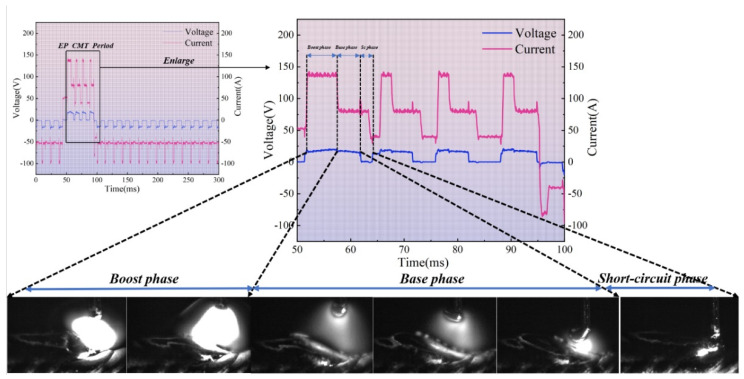
A droplet transition process during the EP stage with a positive–negative polarity ratio of 4:16.

Taking the EP:EN = 4:16 parameter as an example, the current–voltage output is shown in Figure 5. During this phase, the voltage is 0 V and the current decreases to approximately 40 A. Heat generation is primarily resistive and negligible compared to arc heating. High-speed camera imagery reveals that during this phase, droplets formed at the wire tip in the preceding stage contact the molten pool. This phase lasts approximately 5 ms. Within these 5 ms, the wire does not retract immediately upon pool contact during the initial 2 ms. Instead, the droplet at the wire tip forms a liquid bridge under the combined effects of surface tension, electromagnetic contraction force, and gravity, spreading uniformly across the molten pool. During the subsequent 3 ms, wire retraction commences. Initially, the wire tip remains partially adhered to the molten pool due to surface tension. As retraction progresses, the liquid bridge breaks at a certain length, initiating an arc between the wire tip and molten pool, marking the onset of the peak current phase.

During the EP peak current phase, the arc characteristics cause the arc to form beneath the droplet at the wire end and within the molten pool. The cladding layer acts as the cathode, with the cathode spot spontaneously seeking the oxide film near the shielding gas edge, forming a “bell-shaped” arc. This arc is relatively divergent. According to electromagnetic contraction theory, the electromagnetic contraction force of a more dispersed arc exerts a greater and more widespread component force on the area below. As the arc originates beneath the molten droplet at the wire tip, it generates a substantial heating area over a wide region of the molten pool, resulting in high thermal input efficiency. Concurrently, a molten droplet forms at the wire tip and gradually enlarges, a process lasting 2 milliseconds. During the base current phase, the current decreases substantially compared to the peak current phase, reducing the heating effect on the weld pool and lowering thermal input. Concurrently, this phase maintains the size of the droplet as the wire is fed downward until the droplet contacts the weld pool, completing one droplet transition cycle.

**Figure 6:**
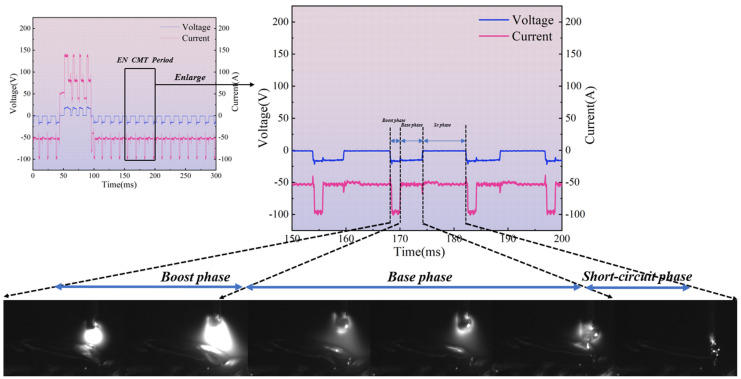
A droplet transition process during the EN stage at a positive–negative polarity ratio of 4:16.

The arc characteristics during the EN phase differ from those in the EP phase. As shown in Figure 6. The wire acts as the cathode, with the arc spontaneously seeking the oxide layer above. The arc forms between the droplet enveloping the wire tip and the molten pool, creating an “enveloping” arc. According to electromagnetic contraction theory, the electromagnetic contraction force of a tightly enveloping arc exerts a smaller and more localised downward component. As the arc originates around the droplet at the wire’s base, this configuration heats the underlying cladding zone inefficiently. Heat predominantly accumulates around the droplet at the wire tip, resulting in lower thermal input to the molten pool area. With the arc concentrated near the droplet, the EN peak current phase significantly heats the wire, accelerating droplet shedding speed compared to the EP peak current phase—approximately 1.5 ms faster. During the EN phase, reduced heat input to the molten pool compared to the EP phase results in a higher cladding zone height. Consequently, wire feed during the EN base current phase contacts the molten pool earlier, shortening this phase duration by approximately 1ms relative to the EP base current phase.

**Figure 7:**
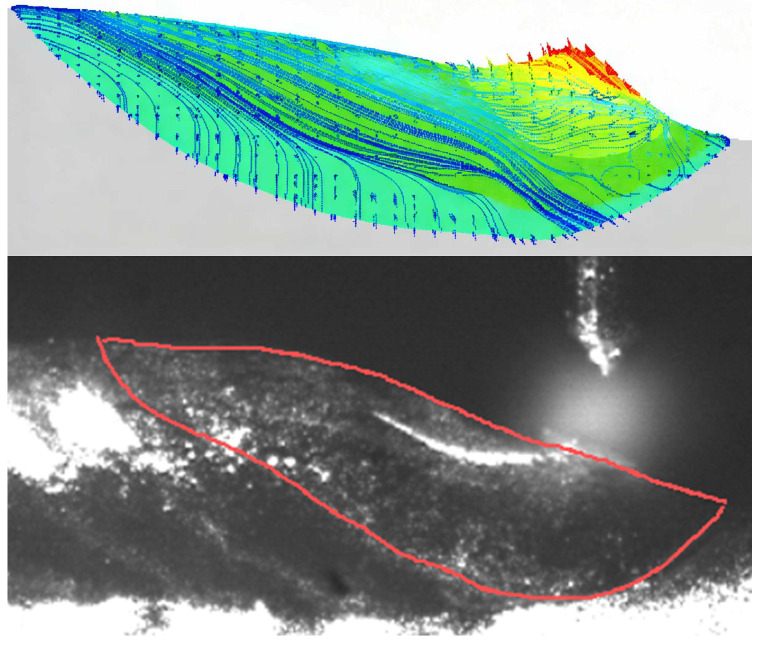
Comparison of simulation results with high-speed camera observations.

The comparison chart of the high-speed camera observation results and the simulation results is shown in Figure 7. The following simulation results illustrate the variation in melt pool length and flow characteristics throughout a complete alternating current cycle when CMT additive deposition occurs at the centre position of the tenth layer:

We can observe that in terms of droplet size: the EN stage exhibits a droplet diameter approximately 0.3 mm larger than the EP stage, as shown in Figure 3. This indicates that the EN stage provides superior heating effects at the wire end compared to the EP stage. Furthermore, compared to the EP stage, the EN stage delivers lower heat input to the molten pool, transferring less heat to the formed component section and accelerating molten pool solidification. Consequently, the EN stage is more conducive to forming narrower and taller clad layers in arc-based additive processes, thereby enhancing cladding efficiency. The heat inputs under the three parameter sets are presented in Table 4:

### 4.1. Analysis of Molten Pool Velocity Under Different EP:EN Parameters for Multi-Layer Single-Pass Additive Manufacturing

**Figure 8:**
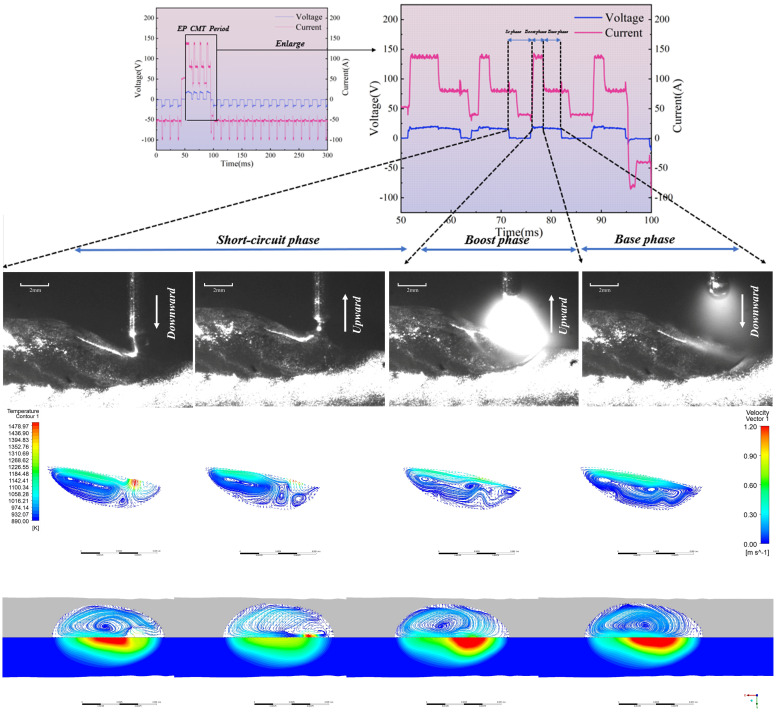
Melt pool evolution during one droplet transition cycle in the EP phase at EP:EN = 4:16.

Figure 8 presents the top-down view of the surface flow field and temperature field during the EP phase, along with the flow field vectors within the molten pool at the XOZ cross-section (along the build direction) and corresponding high-speed camera images for the EP/EN = 4:16 parameters. It can be observed that during the EP short-circuit phase, the droplet at the wire tip impinges upon the molten pool with a certain momentum before transitioning uniformly into the pool. The highest temperatures occur near the droplet, which spreads radially across the molten pool surface. Driven by Marangoni forces, the droplet flows along the pool periphery towards the rear, where it recirculates towards the pool centre and bottom. The highest flow velocity occurs when the droplet first contacts the molten pool and at the rear surface of the molten pool, at approximately 0.6 m/s and 0.5 m/s respectively. During the EP short-circuit retraction phase, the molten pool and the liquid bridge section of the welding wire move upwards. The molten pool near the liquid bridge also moves upwards, reaching a maximum velocity of 0.8 m/s, driving the molten pool surface to flow upwards.

Upon reaching the peak current phase of EP, the upward-moving liquid bridge breaks, causing the entire surface molten pool to flow rearward. Concurrently, the arc generated during peak current exerts significant arc pressure, creating a squeezing effect beneath the molten pool. This induces rearward flow within this region, which converges with the rearward-flowing molten pool at the pool’s midpoint, flowing upward. By the time the EP base current phase stabilises, two vortices form within the molten pool.

**Figure 9:**
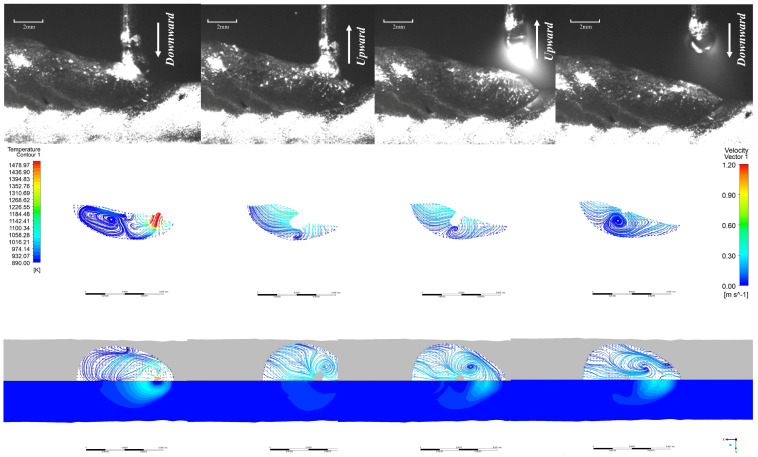
EN phase at EP:EN = 4:16.

The EN short-circuit phase resembles the EP phase, as illustrated in Figure 9. The primary distinction lies in the peak and base current phases where the arc does not diverge. Consequently, the majority of the arc’s energy is directed towards the droplet, resulting in a larger droplet size compared to the EP phase. The range and magnitude of arc pressure acting upon the molten pool surface are reduced, yielding less pronounced effects. Internal flow within the molten pool is minimal, with the maximum flow velocity occurring precisely at the moment the liquid bridge breaks.

**Figure 10:**
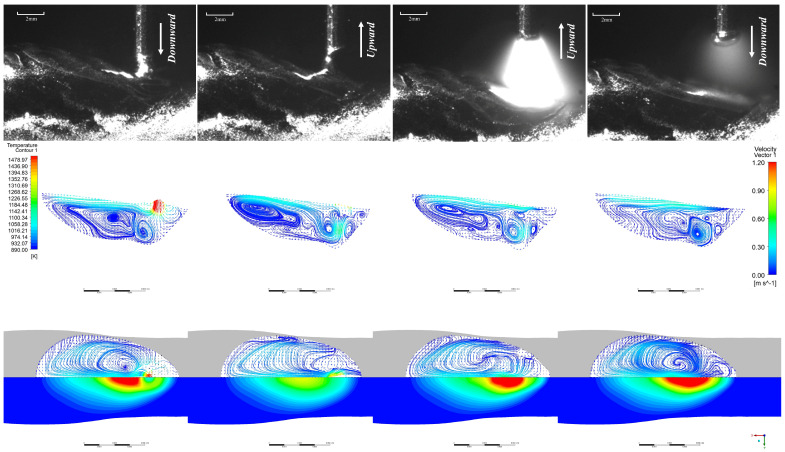
EP stage at EP:EN = 8:12.

Figure 10 presents the top-down view of the melt pool surface flow field and temperature field, along with the flow field vector diagram within the melt pool at the XOZ cross-section (along the additive direction), under the EP/EN = 8:12 parameters. It can be observed that under EP/EN = 8:12, during the EP short-circuit phase, the droplet at the wire end impinges upon the molten pool with a certain momentum, as shown in Figure 10. A droplet-like liquid forms ahead of the molten pool and rapidly plunges into it. Upon contact, the molten pool reaches a peak velocity of 1.2 m/s, after which the droplet transitions uniformly into the pool. Owing to the markedly distinct temperature field at the molten pool surface, the molten metal near the surface region is driven by the Marangoni force as the primary propulsive force, inducing surface flow. It can be observed that the droplet spreads outwards along the molten pool surface, converging with the molten pool flowing back from the rear of the pool before falling behind the droplet. Within the molten pool, the downward motion of the droplet opposes the direction of the returning molten pool beneath it, generating a vortex at the pool’s base. After 0.004 s, the retraction of the welding wire pulls the molten pool region adhering to the droplet upwards. This upward velocity, counter to the initial downward velocity of the droplet, causes turbulent flow beneath the droplet, generating numerous small vortices in the vicinity. The surface velocity of the molten pool reaches approximately 0.4 m/s. At 0.006 s, the welding wire separates from the droplet, initiating an arc. Under the combined effects of arc force compression and Marangoni forces, the surface molten pool flows rearward before recirculating along the pool’s periphery toward the front. The maximum velocity occurs near the arc’s centre, reaching approximately 0.6 m/s. Within the molten pool, having just initiated the arc, the surface arc pressure and Marangoni forces interact with the internal gravitational, buoyant, and electromagnetic forces. The internal environment remains unstable, sustaining numerous small vortices. At 0.010 s, the sustained heating from the base current phase expands the high-temperature zone relative to 0.006 s. Various regions within the molten pool gradually stabilise, with minor vortices diminishing. After 0.011 s, the next cycle commences.

**Figure 11:**
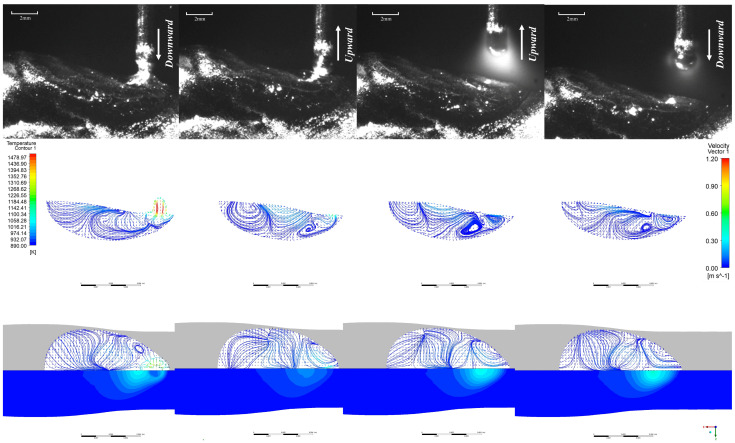
EN stage at EP:EN = 8:12.

**Figure 12:**
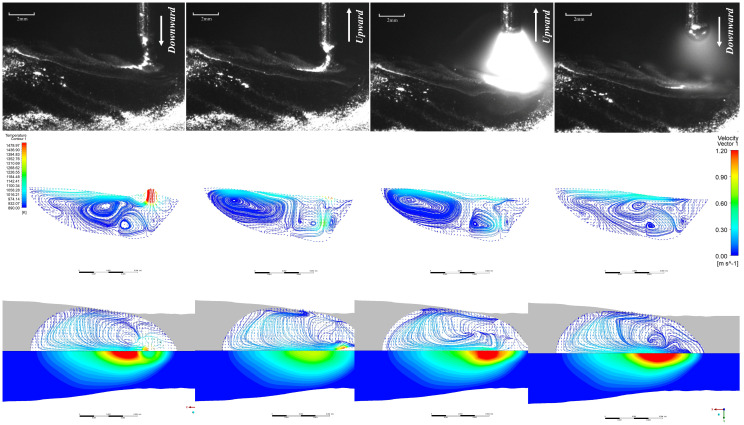
EP phase at EP:EN = 12:8.

**Figure 13:**
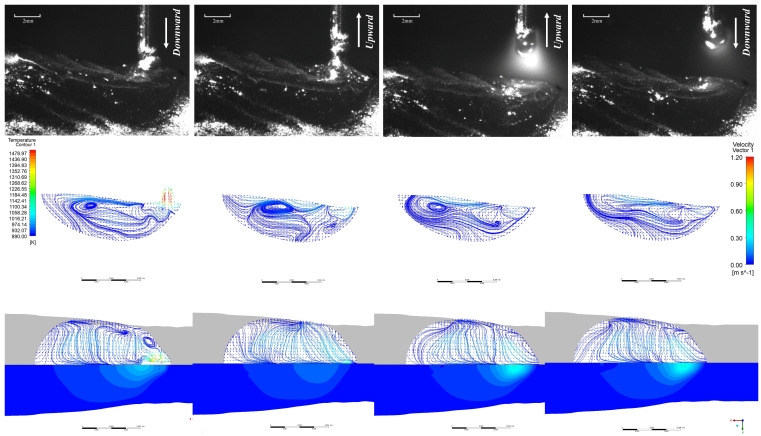
EP:EN = 12:8 during the EN stage.

The EN short-circuit phase commences with droplets contacting the molten pool with a certain momentum. As illustrated in Figure 11, similar to the EP phase, droplet-like liquid forms ahead of the molten pool and enters it. However, due to the arc polarity reversal characteristic of the EN phase, the arc heat is concentrated at the wire end, resulting in larger droplet size—approximately 1.2 times that of the EP phase. The heat input at the pool end is relatively low. It can be observed that the overall pool temperature during the EN phase differs significantly from that of the EP phase, with the pool size being smaller and its fluidity reduced. Consequently, apart from the substantial changes in pool flow velocity caused by droplet impact and retraction during the EN phase, the maximum flow velocity at the pool surface and within the pool does not exceed 0.2 m/s at other times. Furthermore, owing to the low heat input characteristic of the EN stage, heat is less likely to accumulate within the molten pool, leading to faster solidification. Consequently, a pronounced upward accumulation trend is evident throughout the cladding layer. EP:EN=12:8 The parameters are similar to those of 8:12. As shown in Figure 12 and Figure 13.

**Figure 14:**
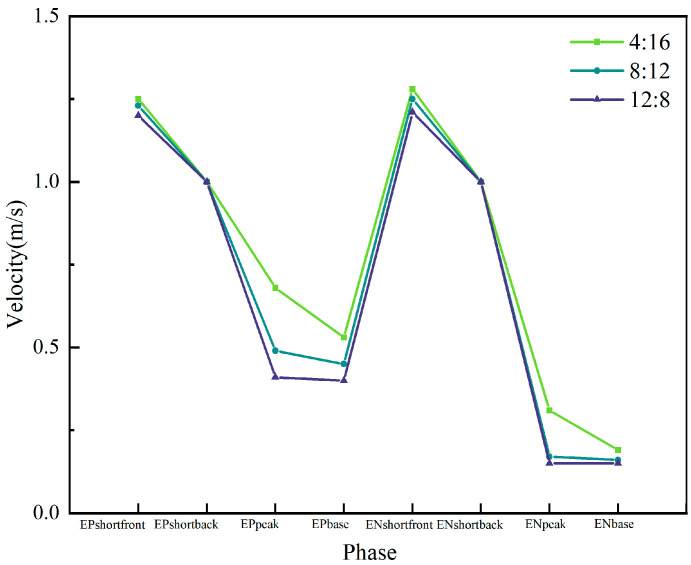
Maximum flow velocities during each phase at different EP:EN ratios.

As shown in Figure 14, the flow velocity at the onset of the EP and EN short-circuit stages is approximately 1.2 m/s across all three parameter sets. During this phase, molten droplets adhere to the wire and move downward with it, resulting in an instantaneous wire velocity of 1.2 m/s. However, the temperature difference between the droplets and the molten pool varies across different parameters, leading to differing Marangoni forces upon contact. The lower molten pool temperature under the EP:EN = 4:16 parameter generates a larger Marangoni force, causing a more significant increase in instantaneous velocity, whereas the higher molten pool temperature at EP:EN = 12:8 results in a smaller increase. By the short-circuit retraction phase, all three parameter sets maintain a consistent velocity of 1 m/s. The overall melt pool velocity is higher during the EP arc ignition phase compared to the EN arc ignition phase. This is attributed to the broader range of arc force influence during the EP phase, resulting in stronger interactions between melt pool liquids and consequently higher velocities. At the peak of the EP arc ignition phase, the maximum velocity occurs at the melt pool surface under the EP:EN = 4:16 parameter, reaching 0.68 m/s. Under EP:EN = 8:12 parameters, the maximum flow velocity similarly occurs at the molten pool surface at 0.49 m/s and similarly at 0.41 m/s under EP:EN = 12:8. This variation arises because, while the arc generated at the wire end during the ignition phase is identical across parameters, the overall temperature distribution within the molten pool and its surface differs significantly. Under EP:EN = 4:16, the greater temperature differential between the droplets and the pool surface results in stronger Marangoni forces at the pool surface, thereby increasing the flow velocity. Under EP:EN = 12:8 parameters, the smaller temperature difference results in weaker Marangoni forces, yielding lower flow velocities. During the EP arc initiation phase, the molten pool gradually stabilises. At the peak phase, the velocity further decreases from the baseline. The maximum velocity is 0.53 m/s under EP:EN = 4:16 parameters, 0.45 m/s under EP:EN = 8:12 parameters, and 0.4 m/s under EP:EN = 12:8 parameters, showing a smaller decrease.

### 4.2. Variation in Molten Pool Dimensions Under Different EP:EN Parameters

**Figure 15:**
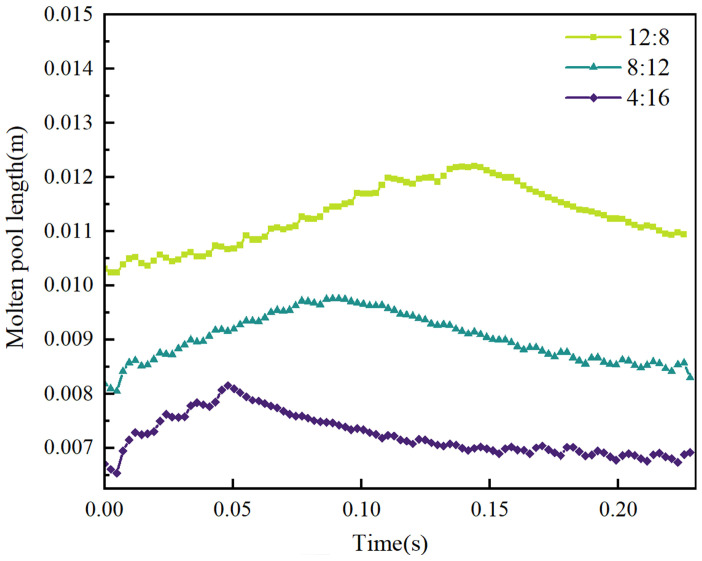
Melt pool length along the X-axis during one VP cycle for different EP:EN ratios in the Z-direction.

The variation in molten pool length within a single EP/EN cycle under three parameters is illustrated in Figure 15. It can be observed that the molten pool length undergoes dynamic changes over time. At EP:EN = 4:16, the molten pool length increases significantly at the onset of the EP phase due to the high peak current period. During the base current phase and short-circuit transition phase, the pool size remains relatively stable owing to the low current levels. After four EP cycles, the molten pool reaches its maximum dimension of 0.0081 m. Subsequently, the EN phase commences. Owing to the relatively low peak and base currents during the EN phase, the molten pool gradually returns to its initial small size after 16 EN phase cycles. Similar variations are observed under other parameter combinations. For EP:EN = 8:12, the molten pool also reaches its maximum size after 8 EP phases, with a peak dimension of approximately 0.0098 m. For EP:EN = 12:8, the maximum size of approximately 0.0121 m was attained after 12 EP cycles. Analysis indicates that during the EP phase, the arc characteristics associated with high thermal input cause the molten pool size to progressively increase. Upon transitioning to the EN phase, the arc characteristics result in lower thermal input, leading the molten pool size to gradually revert to its initial state at the cycle’s commencement.

When EP:EN = 4:16, due to the reduced EP phase duration under these process parameters, the effects of high heat input, high arc pressure, and wide arc range are diminished. Consequently, the thermal input to the molten pool during a complete EP/EN cycle phase is lower, and the arc force’s effect on the molten pool is weaker, resulting in reduced spreading of the molten pool. Therefore, in VP-CMT mode, when performing continuous reciprocating single-pass multi-layer deposition up to approximately the midpoint of the 10th layer, the overall molten pool size is smaller compared to when EP:EN = 8:12 and EP:EN = 12:8. At EP:EN = 12:8, the EP phase is more frequent under these parameters, with greater thermal input, higher arc pressure and range exerting more influence. During a complete EP/EN cycle phase, the thermal input to the molten pool is higher, and the arc force effect on the molten pool is stronger, i.e., the spreading effect on the molten pool is more pronounced. In VP-CMT mode, during continuous reciprocating single-pass multi-layer deposition up to the midpoint of the 10th layer, the overall molten pool exhibits a larger size compared to EP:EN = 4:16 and EP:EN = 8:12. At EP:EN = 8:12, the EP and EN phases achieve relative equilibrium. The eight droplet transition cycles in the EP phase deliver sufficient heat, while the twelve cycles in the EN phase provide effective cooling for the molten pool. The arc forces exerted during both EP and EN phases reach a mutually reinforcing state.

### 4.3. Changes in Molten Pool Dimensions Under Different EP:EN Parameters

**Figure 16:**
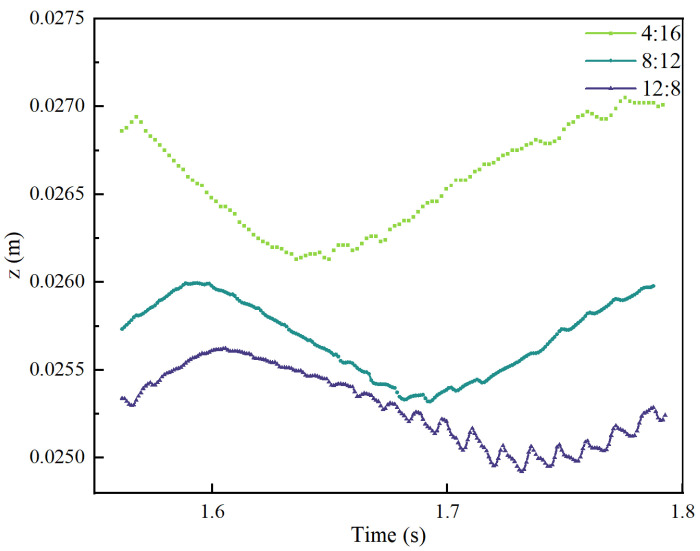
Distribution of Z-axis coordinates at different EP:EN ratios within one VP cycle in the Z direction.

In this study, the minimum z value of the liquid phase at the molten pool surface in simulation results characterises the extent of the lateral flow of the molten pool. As illustrated in Figure 16, the extent of pool flow during the EP and EN phases under three sets of parameters is evident. At identical travel and wire feed rates, the differing average thermal inputs per cycle across the three parameter sets result in variations in pool size and flow state by the tenth layer. Under EP:EN = 4:16, the flow extent during EP and EN phases is as follows: Under EP:EN = 8:12, the flow extent during EP and EN phases is as follows: Under EP:EN = 12:8, the flow extent during EP and EN phases is as follows: A lower number of EP cycles results in reduced flow, making the melt pool less prone to lateral flow. Conversely, an increased number of EP cycles leads to greater flow, causing the melt pool to flow more readily to the sides.

## 5. Conclusions

(1) Molten pool flow behaviour is dominated by Marangoni forces, with significantly higher flow velocities during the EP phase. Simulations indicate that surface flow is primarily driven by Marangoni forces induced by temperature gradients. During the EP phase, arc divergence, high heat input, and extensive arc pressure influence result in intense internal flow, with peak velocities reaching 0.68 m/s. In contrast, the EN phase features a concentrated arc, weaker thermal effects, and lower flow velocities. As the EP proportion increases (4:16 → 12:8), the overall bath temperature rises, the temperature difference between droplets and the bath decreases, and the Marangoni force weakens. Consequently, the peak flow velocity during the EP phase exhibits a decreasing trend.
(2) The molten pool dimensions exhibit periodic dynamic variations, with maximum size positively correlated to the EP proportion. Within a VP-CMT cycle, molten pool length fluctuates cyclically with energy input: high thermal input during the EP phase causes sustained expansion to the cycle’s maximum size; subsequent low thermal input during the EN phase induces cooling, leading to gradual contraction. The thermal accumulation effect is pronounced in multi-layer additive manufacturing, with greater EP proportion resulting in more significant molten pool expansion. Simulations up to the 10th layer showed the maximum molten pool length increasing sequentially with the EP:EN ratio from 4:16, 8:12 to 12:8, reaching 0.0081 m, 0.0098 m and 0.0121 m respectively.
(3) The degree of melt pool flow intensifies with increasing EP proportion, directly influencing the morphology of the cladding layer. The extent of lateral spread is characterised by the height difference between the surface’s lowest point and the underlying layer. The high thermal input and broad arc pressure range during the EP stage significantly enhance the fluidity and spreading capability of the melt pool metal. Consequently, a higher EP ratio (12:8) results in greater flow, producing a wider and flatter clad layer; conversely, a low EP ratio (4:16) exhibits minimal flow, with the clad layer tending to build upwards and exhibiting reduced width. This provides a basis for controlling the width-to-height ratio of the clad layer by adjusting the EP:EN ratio.

## Figures and Tables

**Table 1:** ER5356 aluminium alloy composition.

Grade	Mg	Si	Fe	Mn	Cu	Zn	Cr	Ti	Al
ER5356	5.1	0.04	0.09	0.15	0.01	0.01	0.07	0.08	Allowance

**Table 2:** Composition of 6061 aluminium alloy.

Grade	Mg	Si	Fe	Mn	Cu	Zn	Cr	Ti	Al
6061	1.0	0.6	0.5	0.11	0.24	0.1	0.1	0.035	Allowance

**Table 3:** VP-CMT arc additive manufacturing process parameters.

Wire Feed Speed (m/min)	Travel Speed (mm/s)	Forward–Reverse Polarity Ratio (EP:EN)
4.5	8	4:16
4.5	8	8:12
4.5	8	12:8

**Table 4:** Heat input under three sets of parameters.

Wire Feed Speed (m/min)	Movement Speed (mm/s)	EP:EN	Heat Input (J/mm)
4.5	8	4:16	74.36
4.5	8	8:12	92.51
4.5	8	12:8	107.98

## Data Availability

The original contributions presented in this study are included in the article. Further inquiries can be directed to the corresponding author.
